# Synthesis and Characterization of Pure Ni and Ni-Sn Intermetallic Nanoparticles

**DOI:** 10.1186/s11671-017-1894-2

**Published:** 2017-02-21

**Authors:** A. Yakymovych, H. Ipser

**Affiliations:** 1 Department of Inorganic Chemistry - Functional Materials, Faculty of Chemistry, University of Vienna, Althanstr. 14, 1090 Vienna, Austria; 2 Department of Metal Physics, Faculty of Physics, Ivan Franko National University of Lviv, Kyrylo i Mephodiy str. 8, Lviv, 79005 Ukraine

**Keywords:** Ni-Sn, Nanoparticles, SEM, X-ray diffraction

## Abstract

The present research focused on the synthesis of Ni and Ni-Sn nanoparticles via a chemical reduction method using hydrazine hydrate. The syntheses were performed applying highly purified water or diethylene glycol as solvent. The produced nanoparticles were characterized by scanning electron microscopy and powder X-ray diffraction. The as-synthesized Ni-Sn nanoparticles with nominal starting ratios Ni:Sn = 3:1, 3:2, and 3:4 consisted of different amounts of pure Ni and a low-temperature Ni_3_Sn_2_ phase. It was found that all synthesized nanopowders had a spherical shape with the largest average size for pure Ni and decreasing size for particles containing Sn. X-ray diffraction showed that all synthesized nanoparticles contained Ni and a low-temperature Ni_3_Sn_2_ phase independent of the initial molar ratio; while Ni_3_Sn and Ni_3_Sn_4_ could not be detected.

## Background

Chemical reduction has become a quite popular synthesis method to produce metal nanoparticles (NPs) due to a relatively simple experimental procedure. However, there are several peculiarities involved in the application of this method, major drawbacks being the sometimes relatively large size of the obtained nanoparticles (up to several hundreds of nanometers) and a core–shell structure with a metallic core and an oxide (hydroxide) shell. In order to control the growth of the reduced particles, polyvinylpyrrolidone (PVP) has been widely used as a capping agent. The PVP molecules attach to the metallic nucleus, thereby reducing the surface energy and preventing a further agglomeration. The changes in morphology and particle size of the produced nanoparticles as a function of the added amount of PVP were investigated in several papers. For instance, Ni nanoparticles were produced by Pandey and Manivannan [1] using hydrazine hydrate and nickel chloride in two different molar ratios, i.e., 13:1 and 20:1, as well as three different concentrations of PVP, namely, 0.5, 1.0, and 1.25 g PVP in 99-ml ethylene glycol. A similar variation of the amount of PVP in relation to the other reagents was used by Roshanghias et al. [2], investigating the impact of the added amount of PVP on the size and morphology of the synthesized Sn-based (Sn-Ag-Cu) nanoparticles. A similar study was performed by Bobadilla et al. [3] who tried to control the size and morphology of as-synthesized Ni-Sn nanoparticles by the amount of PVP added. Zhang et al. [4] synthesized Ag nanoparticles by reducing silver nitrate with hydrazine hydrate, controlling the particle size by the PVP/AgNO_3_ molar ratio (up to 2.25). Based on all these results, it can be concluded that increasing the amount of PVP decreases the average size of the as-synthesized metal nanoparticles. It was also shown that, depending on the synthetic conditions, PVP can serve not only as a capping but also as a reducing agent [5].

There are also several reports dealing with the synthesis of bimetallic Ni-Sn nanoparticles via the chemical reduction method. Unfortunately, some of these papers did not provide any structural analysis, and others presented controversial results. For instance, Milanova et al. [6] synthesized Ni-Sn nanoparticles at a mass ratio Ni:Sn = 2:3 (molar ratio approx. 4:3) with a C-foam support using sodium borohydride (NaBH_4_) in an aqueous solution at room temperature. However, it is practically impossible to estimate the average size of the obtained nanoparticles from the presented SEM images, and no diffraction pattern of the Ni-Sn nanoparticles was shown. In a later paper of this group, the same method was used to synthesize Ni-Sn nanoparticles at mass ratios of Ni:Sn = 3:1 and 3:2 (molar ratios approx. 6:1 and 3:1) with and without different supports [7]. The obtained nanopowder was additionally dried in vacuum for 24 h at 373 K which had not been mentioned in Ref. [6]. Furthermore, there is some contradiction between the results shown in Fig. 4 of Ref. [7] and the corresponding text: whereas in the text it is mentioned that the compound Ni_3_Sn is formed in nanopowders with a molar ratio Ni:Sn = 6:1 and the compound Ni_3_Sn_2_ in those with a molar ratio Ni:Sn = 3:1, the figure seems to indicate the opposite. In contrast, Ni-Sn nanoparticles with molar ratios Ni:Sn = 2:1, 1:1, 2:3, and 1:2 using NaBH_4_ as a reducing agent were also synthesized by Liu et al. [8]. The main difference in synthesis compared to the procedure in Refs. [6, 7] was the addition of trisodium citrate (Na_3_C_6_H_5_O_7_) to the mixture of metal ions and the addition of ammonia to adjust the pH value. The samples were dried in vacuum at 343 K. From the XRD patterns, it was concluded that nanopowders with molar ratios Sn:Ni = 2:1 and 1:1 consisted mainly of Sn_6_O_4_(OH)_4_ and pure Sn while nanopowders with molar ratios Sn:Ni = 2:3 and 1:2 contained the compounds “NiSn” (a compound which does not exist in the phase diagram [9]) and Ni_3_Sn as well as pure Sn. According to the presented SEM images, the size of as-synthesized nanoparticles was between 50 and 100 nm. Pure Ni and Sn nanoparticles as well as bimetallic Ni-Sn nanoparticles with initial weight ratios Ni:Sn = 9:1; 3:1; 1:1, and 1:3 (molar ratios 19:1; 6:1; 2:1, and 2:3, respectively) were produced from aqueous solutions using nickel sulfate and tin sulfate as metal precursors and PVP and NaBH_4_ as a capping and reducing agent, respectively, by Dhanapal et al. [10]. According to the provided FESEM analysis, the Ni nanoparticles had a sheet or foam like morphology while the XRD pattern showed a broad diffraction line with three small sub-peaks of lower intensity. In contrast, Sn nanopowder consisted of spherical and mostly agglomerated fine particles, and the XRD pattern showed sharp diffraction lines corresponding to the crystal structure. The surface morphology of bimetallic Ni-Sn nanoparticles changed from a foam-like type for nanopowder with the molar ratio Ni:Sn = 19:1 to an ultrafine spherical-like type for nanopowder with the molar ratio Ni:Sn = 2:3. Based on the XRD patterns, it was suggested that Ni-Sn nanoparticles contained nickel hydroxide and tin hydroxide. The prepared nanopowders, except nano Sn, were annealed afterwards for 3 h at 773 K under N_2_ atmosphere. The XRD analysis of the annealed samples showed that amorphous Ni nanoparticles had transformed into nanocrystalline Ni. The diffraction peaks of nanoparticles with a Ni:Sn molar ratio of 19:1 corresponded also to the Ni fcc structure. XRD patterns of annealed Ni-Sn nanoparticles with initial molar ratio Ni:Sn = 6:1 revealed the formation of the Ni_3_Sn phase besides pure Ni. Annealed nanoparticles with Ni:Sn = 2:1 contained Ni and Ni-Sn phases; while only the Sn phase could be distinguished from the XRD pattern of Ni-Sn nanoparticles with an initial molar ratio Ni:Sn = 2:3.

The major benefits of employing a polyalcohol, i.e., mainly ethylene glycol, as solvent instead of an aqueous solution are:

-A polyalcohol can serve as both solvent and reducing agent [11];
-The nucleation and growth processes of nanoparticles can be controlled through selection of a specific temperature due to the temperature dependence of the reduction power of a polyalcohol [12].

For instance, Bobadilla et al. [3] synthesized Ni-Sn nanoparticles with an initial molar ratio Ni:Sn = 3:1 while the diffraction lines in the XRD pattern of the obtained nanopowder corresponded to the intermetallic compound (IMC) Ni_3_Sn_2_. The solution was firstly heated to 323 K, and after addition of NaBH_4_ refluxed at 473 K for 2.5 h. The produced nanopowder was washed and dried at 373 K for 12 h. The authors concluded that several XRD peaks could be referred to Ni_3_Sn; however, taking into account the very poor signal-to-noise ratio, this appears to be an overly optimistic assumption. Based on the EDS analysis, it was pointed out that as-synthesized Ni-Sn nanopowders consisted of Ni_3_Sn, Ni_3_Sn_2_, and Ni-Sn solid solution. It was also shown that the addition of PVP during synthesis led to a decrease in size of the as-synthesized particles from 9 to 5.5 nm. Based on XRD analysis, combined with TEM and HREM images, authors concluded that the nanoparticles were of polycrystalline nature, formed by the assembly of smaller particles with a size up to 4 nm. Fourier Transform Infrared spectroscopy analysis (FTIR) of the samples indicated that a liquid-like PVP layer encapsulated the NPs preventing particle-particle contact and thus the aggregation of the nanoparticles. Microstructure, phase transformation, and reduction behavior of Ni and Ni-Sn nanoparticles with initial molar ratio Ni:Sn = 3:1, produced by the same route and supported by CeO_2_-MgO-Al_2_O_3_, were investigated in a later paper of the same group [13]. A very similar synthesis route was used by Shah et al. [14] to produce Ni-Sn nanoparticles with nominal molar ratios Ni:Sn = 3:1, 3:2, and 1:1. The TEM micrographs showed both well-dispersed nanoparticles and elongated particles with average sizes of 4.5, 8.0, and 7.2 nm for Ni_74_Sn_26_, Ni_59_Sn_41_, and Ni_50_Sn_50_ nanoparticles (at.%; compositions were determined by EDX), respectively. The authors also noted that the EDX analysis of the agglomerated particles indicated higher amounts of Sn compared to well-dispersed nanoparticles from which they concluded that the produced nanopowders contained particles with various compositions making them quite heterogeneous. In addition, XRD patterns of Ni-Sn nanoparticles showed two broad peaks with low intensity for compositions Ni:Sn = 3:2 and 1:1, similar to those for nanoparticles with initial a molar ratio Ni:Sn = 3:1 in Ref. [3]. In contrast, the XRD pattern for nanopowder with a molar ratio Ni:Sn = 3:1 showed one broad peak at 2*θ* = 43.05^0^ which is typical for amorphous materials.

Based on the abovementioned literature, it can be concluded that as-synthesized Ni-Sn nanopowders produced by the polyol method have an amorphous structure and are relatively small in size up to 10 nm. At the same time, they can contain particles with various concentrations of the components different from the initial concentration. It should be noted, that amorphous Ni-Sn nanoparticles were also obtained by dissolving metal precursor salts in absolute alcohol, mixing with sodium citrate solution, and reduction with KBH_4_ [15]. The SEM analysis of as-synthesized Ni-Sn nanopowder showed agglomerated particles 0.5–20 μm in size.

In all the studies discussed above, except Ref. [15], NaBH_4_ was used as reducing agent. On the other hand, hydrazine hydrate has also been widely used as reducing agent in the synthesis of bimetallic nanoparticles, for instance, to produce Co-Sn [16], Fe-Ni [17, 18], and Ag-Ni [19] nanoparticles, via a hydrothermal reaction. This fact, together with the manifested differences of structural data in the literature, motivated us to study the structure and morphology of as-synthesized Ni-Sn nanoparticles (molar ration Ni:Sn = 1:3, 2:3, and 4:3) obtained via the chemical reduction method using hydrazine hydrate. The synthesis was performed applying two different solvents, namely, highly purified Milli-Q water (water purified using a Millipore Milli-Q® lab water system) and diethylene glycol (DEG). The as-synthesized nanoparticles were characterized using powder XRD and SEM analysis.

## Methods

### Chemicals and Materials

All reagents were commercially available and used as received without further purification. Pure Ni and bimetallic Ni-Sn nanoparticles were produced via a hydrazine reduction method by employing sodium hydroxide (NaOH; Merck) as well as hydrazine hydrate (N_2_H_4_ · H_2_O; Sigma-Aldrich) and polyvinylpyrrolidone (PVP; Alfa-Aesar) as the reducing agent and surfactant agent, respectively. The stoichiometric amounts of nickel chloride (NiCl_2_; Loba-Chemie) and tin(II) 2-ethylhexanoate (C_16_H_30_O_4_Sn; Alfa-Aesar) were dissolved in purified water (Milli-Q water; produced by a Milli-Q SP Ultra-Pure-Water Purification System) or diethylene glycol (DEG; Alfa-Aesar) as the metal precursors.

### Synthesis

At the beginning, 0.6-g PVP was added into a flask with 120 mL of solvent under continuous stirring. Appropriate amounts of nickel chloride and tin(II) 2-ethylhexanoate were dissolved in the solvent at a temperature of 343 K to produce 0.5-g metal nanopowder. Subsequently, 4.0-g sodium hydroxide, dissolved separately in 10 mL of Milli-Q water, and 60 mL of hydrazine hydrate were added to the solution of the metal precursors under rapid stirring. The color of the solution changed from initially clear green to turbid green by the addition of sodium hydroxide, and to blue by the addition of hydrazine hydrate. After a certain period of time, the color turned to black, indicating the formation of metal nanoparticles (route 1). In the case of using diethylene glycol as a solvent, the solution was heated from 343 to 473 K and refluxed for circa 30 min followed by natural cooling to room temperature (route 2). The nanopowder was recovered by centrifugation at 4000 rpm for 30 min, rinsed several times with absolute ethanol, acetone, and distilled water to remove impurities. The filtered nanopowder was finally dried in vacuum for one day at room temperature.

#### Characterization of Ni and Ni-Sn Nanoparticles

The particle size and morphology of the precipitated nanoparticles were examined using a scanning electron microscope (ESEM, Zeiss Supra 55VP). A quantitative analysis of the nanoparticles was performed using Co for the energy calibration of the energy-dispersive X-ray (EDX) detector signal. Samples were prepared by dropping a few drops of a dispersion of the nanoparticles in ethanol on a polished Si wafer. Crystallographic structure and crystallite size of the produced nanopowders were identified using X-ray powder diffraction (XRD, Bruker D8 Advance). Rietveld refinement of XRD patterns was performed with the Topas 3 software.

## Results and Discussion

### Structure and Morphology

**Fig. 1:**
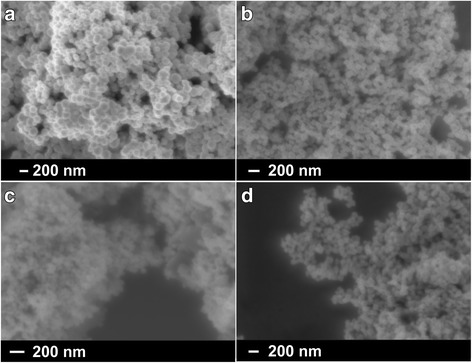
SEM images of nano Ni (**a**) and bimetallic Ni-Sn nanoparticles with molar ratios Ni:Sn = 3:1 (**b**); 3:2 (**c**) and 3:4 (**d**) synthesized via first route

**Fig. 2:**
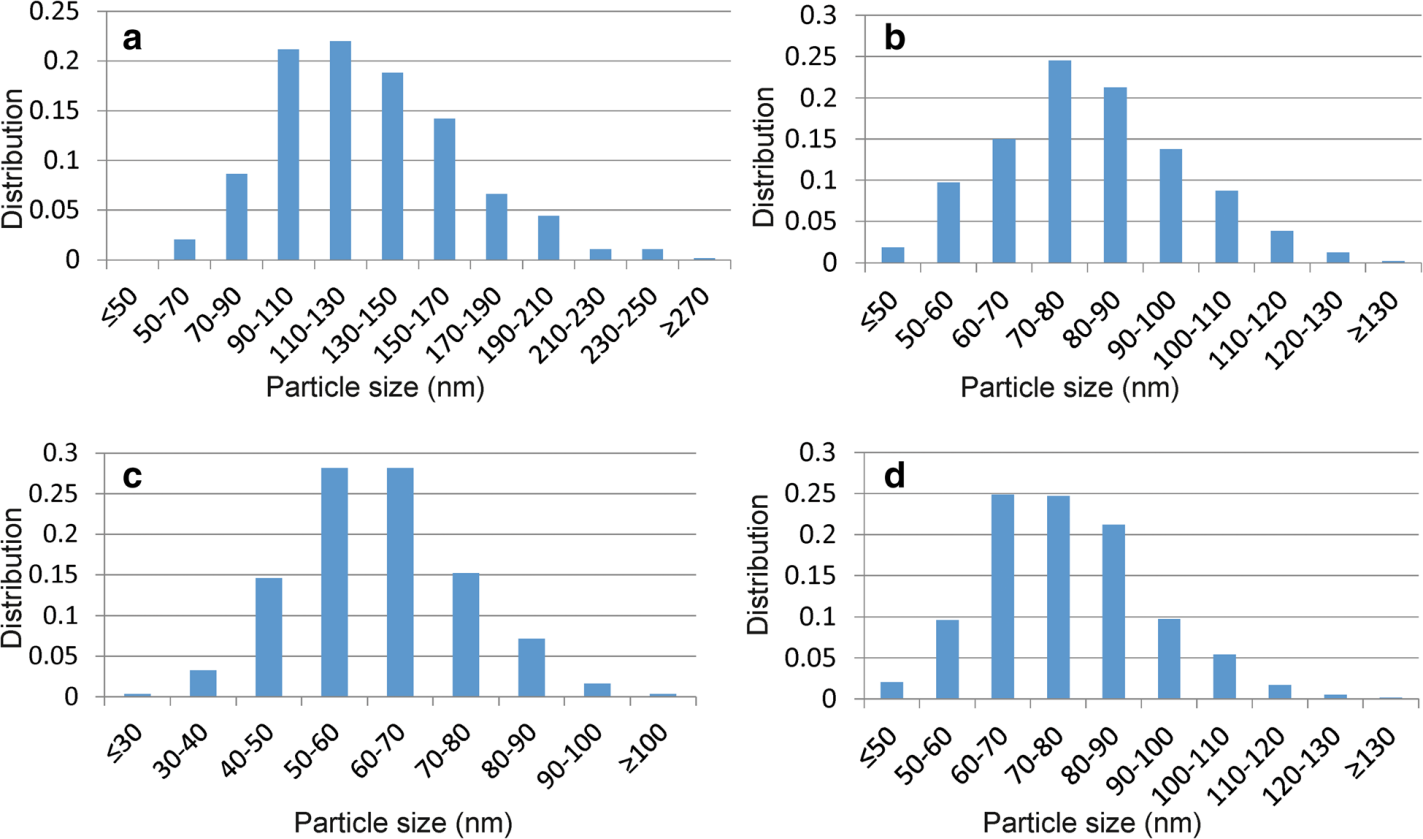
Particle size distribution of nano Ni (**a**) and bimetallic Ni-Sn nanoparticles with molar ratios Ni:Sn = 3:1 (**b**); 3:2 (**c**) and 3:4 (**d**) synthesized via first route

In a first experiment, Ni and Ni-Sn nanoparticles were produced employing Milli-Q water as a solvent. Fig. 1 shows SEM images of the corresponding Ni and Ni-Sn nanopowders. The Ni and Ni-Sn nanoparticles are characterized by a spherical morphology with a decrease in size with the addition of Sn. For instance, the average particle size of as-synthesized nano Ni is equal to 131 nm and decreases to 81, 63, and 77 nm for nano Ni:Sn = 3:1, 3:2, and 3:4, respectively (Fig. 2). The average size distribution of the produced nanoparticles was estimated using several SEM images suggesting sphere-like morphology of the produced nanoparticles.

**Fig. 3:**
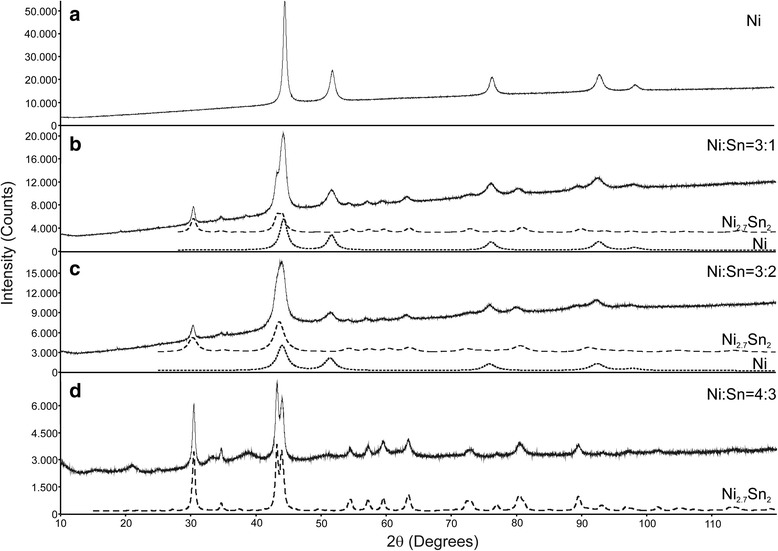
XRD patterns of Ni and Ni-Sn nanoparticles synthesized via first route. **a**, **b**, **c** and **d**

The peaks of the XRD pattern for nano Ni corresponded to the fcc Ni structure (Fig. 3). The pattern of the Ni-Sn nanoparticles with an initial ratio Ni:Sn = 3:4 could be readily indexed to a low-temperature Ni_3_Sn_2_-type orthorhombic structure, namely, the Ni_2.7_Sn_2_ phase [20]. The analysis of the XRD patterns of the other Ni-Sn nanopowders showed evidence of the same two phases, i.e., Ni and Ni_2.7_Sn_2_, in different ratios. Based on these results, it is suggested that with the described synthesis route only the Ni_3_Sn_2_ phase is formed as the most stable intermetallic phase in the Ni-Sn system. The attempt to synthesize pure Sn nanoparticles through the same route failed surprisingly. An initial nucleation of Ni coated with Sn in a second reaction is suggested. This is in agreement with literature data [14].

**Fig. 4:**
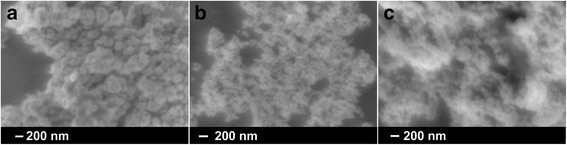
SEM images of bimetallic Ni-Sn nanoparticles with molar ratios Ni:Sn = 3:1 (**a**); 3:2 (**b**) and 3:4 (**c**) synthesized via second route

**Fig. 5:**
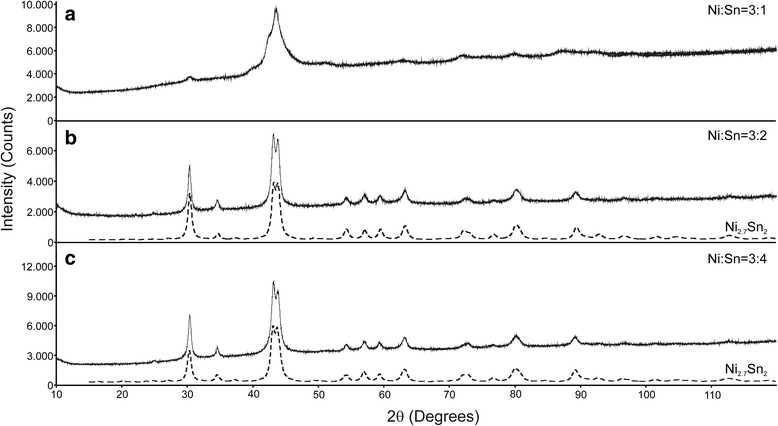
XRD patterns of Ni-Sn nanoparticles synthesized via second route. **a**, **b** and **c**

The SEM images of the Ni-Sn nanoparticles produced via the second route, shown in Fig. 4 indicate a sphere-like morphology for those with an initial ratio Ni:Sn = 3:2 while nanoparticles with initial ratios Ni:Sn = 3:1 and 3:4 show a mixture of irregular and spherical shape. Furthermore, these nanoparticles were larger in size compared to the ones prepared via the first route. It is assumed that the elongated NPs in Fig. 4a consist of smaller and mostly amorphous nanoparticles sticking together as indicated by the corresponding X-ray pattern (Fig. 5a). The other nanopowders produced via the second route are obviously characterized by the low-temperature Ni_3_Sn_2_-type orthorhombic structure. The X-ray analysis of the produced NPs with the initial molar ratio Ni:Sn = 3:2 did not show any peaks related to pure Ni (Fig. 5b) similar to those with an initial molar ratio Ni:Sn = 3:4.

The abovementioned heterogeneous nucleation leads to the production of nanoparticles with local concentration gradients compared to the initial composition, a fact mentioned also in Refs. [3, 7, 8, 14]. For instance, based on the EDX analysis it was estimated that the concentration of Ni in the Ni-Sn NPs produced via the second route with initial molar ratios Ni:Sn = 3:1, 3:2, and 3:4 changes in the range 67–82 at.%, 55–62 at.%, and 56–68 at% Ni, correspondingly.

It should also be noted that no obvious oxidation peaks were found in the XRD patterns of the as-synthesized nanopowders. The expected formation of a core–shell structure of the produced nanoparticles suggested the formation of a thin Sn-containing shell (tin oxide or tin hydroxide). However, a corresponding proof would require an additional TEM analysis, a method which was unfortunately not available during this study.

The available literature data relating to the synthesis of Ni-Sn nanoparticles are presented in Table 1. The differences in the various resulting phases should be seen in connection with the differences in both the synthesis procedure and the salts employed as metal precursors. The present experimental results are in agreement with Refs. [7, 14] in that respect that Ni_3_Sn_2_ seems to be the most stable intermetallic phase which forms in Ni-Sn NPs with a nominal composition range 43-75 at.% Ni; in contrast, neither Ni_3_Sn nor Ni_3_Sn_4_ could be identified in any nanoparticles in the present research.

**Table 1:** Literature data and own experimental results concerning nominal compositions and observed phases in the produced Ni-Sn NPs together with temperature of synthesis

Nominal ratio (mole fraction)		Synthesistemperature	Observed phases			Ref.
Ni	Sn					
3	1	Room temperature	Ni_3_Sn_2_			[7]
6	1		Ni_3_Sn			
2	1	Room temperature	Sn_6_O_4_(OH)_4_	Sn		[8]
1	1		Sn_6_O_4_(OH)_4_	Sn		
2	3		NiSn	Ni_3_Sn	Sn	
1	2		NiSn	Ni_3_Sn	Sn	
19	1	Room temperature	unknown			[10]
6	1		Ni(OH)_2_ Ni_3_Sn^a^			
2	1		Ni^a^	Sn^a^		
2	3		Sn(OH)_2_ Sn^a^			
3	1	Solution prepared at 323 K and refluxed at 470 K for 2.5 h	Ni_3_Sn	Ni_3_Sn_2_		[14]
3	2		Ni_3_Sn	Ni_3_Sn_2_		
1	1		Ni_3_Sn	Ni_3_Sn_2_		
3	1		Ni_2.7_Sn_2_			This paper
3	2	343 K	Ni_2.7_Sn_2_	Ni		
3	4		Ni_2.7_Sn_2_	Ni		
3	1	Solution prepared at 323 K and refluxed at 473 K for 0.5 h	Not indexed			This paper
3	2		Ni_2.7_Sn_2_			
3	4		Ni_2.7_Sn_2_			

^a^Afterwards annealed under N_2_ atmosphere for 3 h at 773 K

Based on the obtained results, it must be concluded that the synthesis of Ni-Sn NPs via the employed method occurs in several steps starting with the initial reduction of Ni. In a second step, Sn is reduced forming a shell around the Ni particles. In the following, the observed Ni_2.7_Sn_2_ phase is formed as a result of their chemical interaction. Depending on the size of the Ni nucleus, it can be either partly or completely transformed into this intermetallic phase. It is also assumed that, in agreement with literature results [2], a thin Sn-oxide or Sn-hydroxide shell is formed protecting the synthesized NPs from further oxidation.

Furthermore, Ni-Sn NPs synthesized via the first route consisted partly of pure Ni which caused them to be ferromagnetic. Such a magnetic behavior of nanoparticles synthesized with an initial molar ratio Ni:Sn = 3:1 was also indicated by Bobadilla et al. [3]. Therefore, such nanoparticles could be of interest for industrial applications as possible bimetallic ferromagnetic materials in nano-sized form. It should also be noted that an increase in the synthesis temperature led to growth and agglomeration of the produced Ni-Sn nanoparticles.

## Conclusions

Ni and bimetallic Ni-Sn nanoparticles were prepared via a chemical reduction method using water and diethylene glycol as medium. DEG and PVP were employed as reducing agent and surfactant, respectively. The SEM images showed a spatial distribution of the as-synthesized nanoparticles with a lower average size for an initial molar ratio Ni:Sn = 3:2. Based on SEM and X-ray analysis, it is assumed that Ni-Sn nanoparticles synthesized in Milli-Q water medium at 343 K contain a Ni/Ni_2.7_Sn_2_ nucleus and a Sn oxide (hydroxide) shell independent of the initial ratio Ni:Sn. The Ni-Sn nanoparticles synthesized in DEG at 473 K were larger and agglomerated in particles of irregular shape. They consisted again mostly of the low-temperature Ni_2.7_Sn_2_ phase.

## Abbreviations

DEG: Diethylene glycol
IMP: Intermetallic phase
Milli-Q water: Water produced by a Milli-Q SP Ultra-Pure-Water Purification System
NPs: Nanoparticles
PVP: Polyvinylpyrrolidone
SEM: Scanning electron microscopy
TEM: Transmission electron microscopy
XRD: X-ray diffraction

## References

[1] Pandey A, Manivannan R (2015). Chemical reduction technique for the synthesis of nickel nanoparticles. Int J Eng Res Appl.

[2] Roshanghias A, Yakymovych A, Bernardi J, Ipser H (2015). Synthesis and thermal behavior of tin-based alloy (Sn-Ag-Cu) nanoparticles. Nanoscale.

[3] Bobadilla LF, Garcia C, Delgado JJ, Sanz O, Romero-Sarria F, Centeno MA, Odriozola JA (2012). Influence of PVP in magnetic properties of NiSn nanoparticles prepared by polyol method. J Magn Magn Mater.

[4] Zhang ZT, Zhao B, Hu LM (1996). PVP protective mechanism of ultrafine silver powder synthesized by chemical reduction processes. J Solid State Chem.

[5] Koczkur KM, Mourdikoudis S, Polavarapu L, Skrabalak SE (2015). Polyvinylpyrrolidone (PVP) in nanoparticle synthesis. Dalton.

[6] Milanova V, Petrov T, Denev I, Markova I (2013). Nanocomposites based on intermetallic nanoparticles template synthesized using different supports. J Chem Techn Metall.

[7] Milanova V, Petrov T, Chauvet O, Markova I (2014). Study of carbon-based nanocomposites with intermetallic (Co-Sn, Ni-Sn) nanoparticles. Rev Adv Mater Sci.

[8] Liu GY, Huang ZL, Yi ZZ, Sun LD, Sun HY (2013). Sn-Ni nano particle prepared by a chemical reduction method. Adv Mater Res.

[9] Schmetterer C, Flandorfer H, Richter KW, Saeed U, Kauffman M, Roussel P, Ipser H (2007). A new investigation of the system Ni-Sn. Intermetallics.

[10] Dhanapal K, Narayanan V, Stephen A (2016). Influence of Sn on the magnetic ordering of Ni-Sn alloy synthesized using chemical reduction method. J Magn Magn Mater.

[11] Pacioni NL, Borsarelli CD, Rey V, Veglia AV (2015) Synthetic routes for the preparation of silver nanoparticles. A mechanistic perspective. In: Alarcon EI, Griffith M, Udekwu KI (Eds.) Silver Nanoparticle Applications. In the Fabrication and Design of Medical and Biosensing Devices. London: Springer International Publishing, pp. 13-46.

[12] Wiley B, Sun YG, Mayers B, Xia YN (2005). Shape-controlled synthesis of metal nanostructures: the case of silver. Chem Eur J.

[13] Bobadilla LF, Palma S, Ivanova S, Dominguez MI, Romero-Sarria F, Centeno MA, Odriozola JA (2013). Steam reforming of methanol over supported Ni and Ni-Sn nanoparticles. Int J Hydrogen Energ.

[14] Shah M, Guo QX, Fu Y (2015). The colloidal synthesis of unsupported nickel-tin bimetallic nanoparticles with tunable composition that have high activity for the reduction of nitroarenes. Catal Commun.

[15] Dong QF, Wu CZ, Jin MG, Huang ZC, Zheng MS, You JK, Lin ZG (2004). Preparation and performance of nickel-tin alloys used as anodes for lithium-ion battery. Solid State Ionics.

[16] Yi Z, Tian X, Han QG, Lian JS, Wu YM, Wang LM (2016). Synthesis of polygonal Co_3_Sn_2_ nanostructure with enhanced magnetic properties. RSC Adv.

[17] Yuan ML, Tao JH, Yu L, Song C, Qiu GZ, Li Y, Xu ZH (2011). Synthesis and magnetic properties of Fe-Ni alloy nanoparticles obtained by hydrothermal reaction. Adv Mater Res.

[18] Liao QL, Tannenbaum R, Wang ZL (2006). Synthesis of FeNi_3_ alloyed nanoparticles by hydrothermal reduction. J Phys Chem B.

[19] Sridharan K, Endo T, Cho SG, Jongryoul K, Park TJ, Philip R (2013). Single step synthesis and optical limiting properties of Ni-Ag and Fe-Ag bimetallic nanoparticles. Opt Mater.

[20] Fjellvag H, Kjekshus A (1986) Structural properties of Co_3_Sn_2_, Ni_3_, Sn_2_ and some ternary derivatives. Acta Chem Scand Ser A: Phys Inorg Chem 40:23-3

